# Postoperative Nomogram for Predicting Cancer-Specific and Overall Survival among Patients with Medullary Thyroid Cancer

**DOI:** 10.1155/2020/8888677

**Published:** 2020-11-22

**Authors:** Li Chen, Yizeng Wang, Ke Zhao, Yuyun Wang, Xianghui He

**Affiliations:** Department of General Surgery, Tianjin Medical University General Hospital, Tianjin Medical University, Tianjin, China

## Abstract

**Background:**

Medullary thyroid carcinoma (MTC) accounts for 1%–2% of thyroid cancer in the United States based on the latest Surveillance, Epidemiology, and End Results (SEER) data, and this study aimed to construct a comprehensive predictive nomogram based on various clinical variables in MTC patients who underwent total thyroidectomy and neck lymph nodes dissection.

**Methods:**

Data regarding 1,237 MTC patients who underwent total thyroidectomy and neck lymph nodes dissection from 2004 to 2015 were obtained from the SEER database. Univariate and multivariate Cox regression analyses were used to screen for meaningful independent predictors. These independent factors were used to construct a nomogram model, a survival prognostication tool for 3- and 5-year overall survival, and cancer-specific survival among these MTC patients.

**Result:**

A total of 1,237 patients enrolled from the SEER database were randomly divided into the training group (*n* = 867) and the test group (*n* = 370). Univariate and multivariate Cox regression analyses were used to identify meaningful independent prognostic factors (*P* < 0.05). Tumor size, age, metastasis status, and LNR were selected as independent predictors of overall survival (OS) and cancer-specific survival (CSS). Finally, two nomograms were developed, and the predicted C-index of overall survival (OS) and cancer-specific survival (CSS) rate in the training group was 0.828 and 0.904, respectively. The predicted C-index of overall survival (OS) and cancer-specific survival (CSS) rate in the test group was 0.813 and 0.828.

**Conclusion:**

Nomograms constructed by using various clinical variables can make more comprehensive and accurate predictions for MTC patients who underwent total thyroidectomy and neck lymph nodes. These predictive nomograms help identify postoperative high-risk MTC patients and facilitate patient counseling on clinical prognosis and follow-up.

## 1. Introduction

Medullary thyroid carcinoma (MTC) is a neuroendocrine malignant tumor derived from parafollicular cells, accounting for 1%–2% of thyroid cancer in the United States based on the latest Surveillance, Epidemiology, and End Results (SEER) data [1]. 25% of MTCs are of hereditary origin, which is related to the RET proto-oncogene, most occurs as part of multiple endocrine neoplasia (MEN) 2 syndrome, and the remaining occur as sporadic forms [2]. The guidelines of the National Comprehensive Cancer Network (NCCN) and the American Thyroid Association (ATA) recommend total thyroidectomy and varied levels of lymphadenectomy for MTC patients. Total thyroidectomy and bilateral central neck dissection are the standard surgical procedures for MTC in sporadic or hereditary form. Patients with primary tumor size greater than 1 cm or central lymph nodes metastasis may be considered for modified ipsilateral radical cervical lymphadenectomy [1]. MTC accounts for 14% of all thyroid cancer-related deaths, and the 10-year survival rate of patients whose tumors are limited to the glands is 95.6%, its 10-year survival rate drops to 75.5% when cervical lymph node metastasis exists; patients with distant metastases had the worst prognosis and only 40% of patients can survive for 10 years [3]. The current prognosis evaluation depends on the American Cancer Society (AJCC) TNM staging system, of which other variables that may be significant for determining the outcome of individual patients are not considered. Ho et al. proposed a nomogram model combined seven indicators, including postoperative calcitonin, vascular infiltration (VI), clinical TNM status, age, and gender to predict the survival prognosis of MTC with a C-index of 0.77 in a small sample size [4]. The Surveillance, Epidemiology, and End Results (SEER) is a public database collecting cancer diagnosis, treatment, and survival data for approximately 30% of the US population and provides researchers with enough clinical information for free [5, 6]. Nomogram is a scientific tool which is convenient for clinicians to use two or more variables to estimate clinical events and has been applied in the prognostic system of many malignant tumors [7]. In this study, we assessed MTC patients who underwent total thyroidectomy and neck lymph nodes dissection registered between 2004 and 2015 in the SEER database and developed validated nomograms for overall survival (OS) and cancer-specific survival (CSS) of MTC patients.

## 2. Materials and Methods

### 2.1. Patients and Data Collection

**Figure 1:**
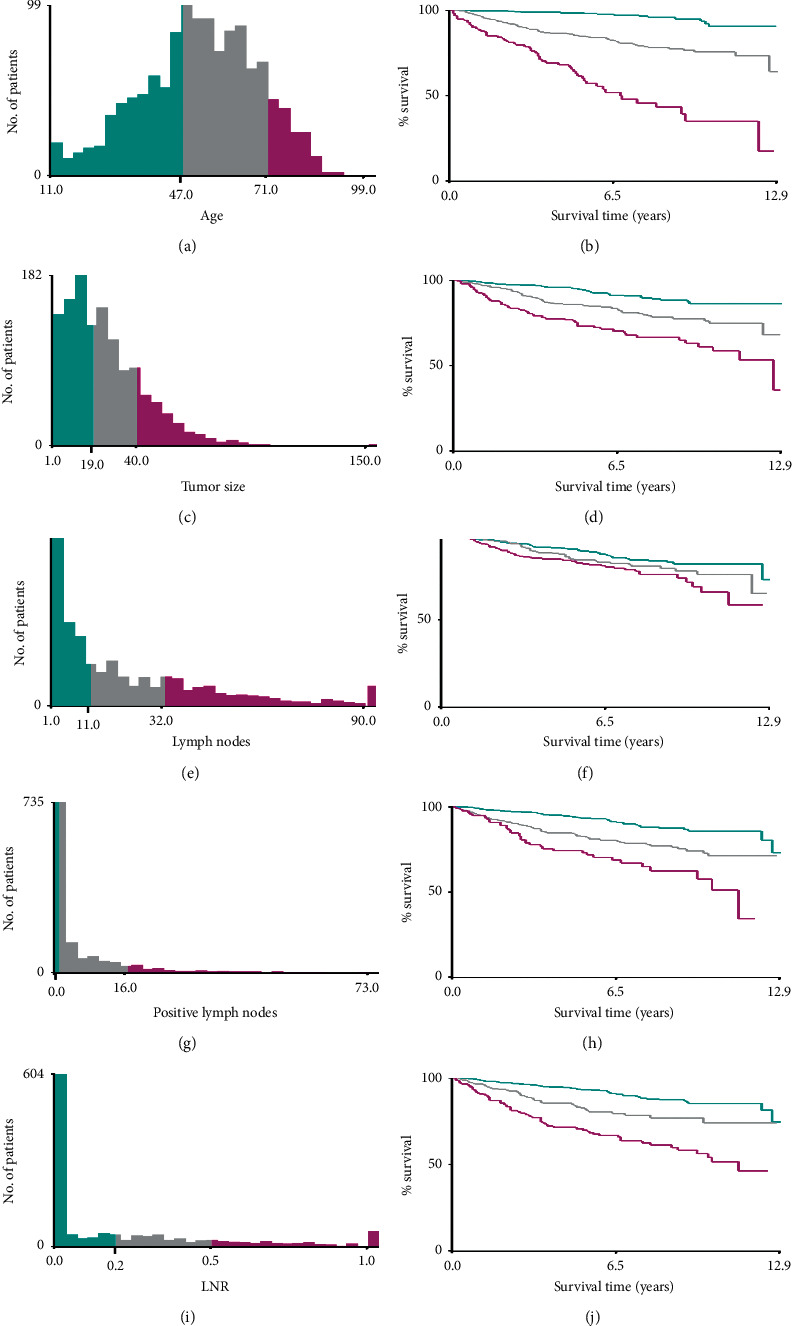
Identification of optimal cutoff values of age (a, b), tumor size (c, d), the number of regional lymph nodes by pathological findings (e, f), pLNs (g, h), and LNR (i, j) via X-tile software analysis. Optimal cutoff values of age were identified as 47 and 71 years based on overall survival. Optimal cutoff values of tumor size were identified as 19 mm and 40 mm based on overall survival. Optimal cutoff values of the number of regional lymph nodes by pathological findings were identified as 11 and 32 based on overall survival. The optimal cutoff values of pLNs were 1 and 16 based on overall survival. The optimal cutoff values of LNR were 20% and 50% based on overall survival.

The data were extracted from the SEER database using the SEER^*∗*^Stat software (version 8.3.6; National Cancer Institute, USA). The patients were limited to being diagnosed with MTC and underwent total thyroidectomy and neck lymph nodes dissection between January 1, 2004, and December 31, 2015. We extracted the following clinical information from the database: age at diagnosis, race (black, white, and others), sex, year of diagnosis, histologic type, clinical TNM stage, tumor size, tumor extension, number of nodules, surgery of primary site, number of removal lymph nodes, the number of regional lymph nodes by pathological findings, the number of positive lymph nodes (pLNs), survival time, cause of death, and survival status, and based on the regional lymph nodes by pathological findings and the numbers of positive lymph nodes (pLNs) of each patient, we calculated the lymph node ratio (LNR). The exclusion criteria were set as follows after obtaining data: (1) patients with missing or unknown clinical information, (2) patients with survival time less than 1 month, and (3) patients without local lymph node dissection. Finally, data of 1,237 patients were collected from the SEER database based on the inclusion and exclusion criteria. X-tile software was used to find out the optimal cutoff values for age, tumor size, the number of regional lymph nodes by pathological findings, the number of pLNs, and LNR (Figure 1). The optimal cutoff values for age were 47 and 71 years; the optimal cutoff values of tumor size were 19 and 40 mm, the optimal cutoff values of the number of regional lymph nodes by pathological findings were 11 and 32, the optimal cutoff values of the number of positive lymph nodes (pLNs) were 1 and 16, and the optimal cutoff values of lymph node ratio (LNR) were 20% and 50%. Clinical staging is based on the 8th edition of the AJCC staging manual. We use stratified sampling to divide 1,237 patients into a training cohort (*n* = 867) and a test cohort (*n* = 370) in the ratio of 7 : 3. The training cohort is used for model construction and the test cohort for external verification. The endpoints of this study are overall survival (OS) and cancer-specific survival (CCS); OS refers to the period from the time of surgery to the death of any cause or the date of the last follow-up, while CSS was calculated from the time of operation to the date of cancer-related death or the time of last follow-up [8]. We calculated the 3-year and 5-year survival rates of OS and CSS at the same time.

### 2.2. Statistical Analysis

Summary statistics tables are used to describe demographic and clinical characteristics of the training cohort and test cohort, and data of continuous and categorical variables are presented as frequencies with percentages using Word 2016 for Windows. Survival time was defined as the time (in months) from surgery to death, last follow-up, or December 31, 2015. The optimal cutoff values of age, tumor size, the number of regional lymph nodes by pathological findings, pLNs, and LNR were evaluated by using the X-tile software as described previously. Univariate Cox regression analysis was applied to evaluate independent survival-related factors for overall survival (OS) and cancer-specific survival (CSS) in the retrospective data. In a multivariate analysis, the Cox proportional hazards regression model was applied for the categorical variables identified as significant in the univariate Cox regression analysis. The intensity of the association between each predicted categorical variables and survival was expressed as a hazard ratio (HR), and 95% confidence intervals (95% CI) were calculated at the same time. The Cox regression analysis was performed by using Statistical Product and Service Solutions (SPSS) version 26.0, and *P* values <0.05 were considered statistically significant. The nomograms were internally and externally validated in training and test cohorts, respectively, Harrell's concordance index (C-index), receiver operating characteristic curve (ROC), and area under the ROC curve (AUC) were used to evaluate the exact prognostic performance of nomograms; the values of C-index and AUC closer to 1 implied a better predictive accuracy. The verification curve can reflect the consistency between the prediction and actual nomograms. The C-index, ROC, nomograms, and the verification curve of the training cohort were formulated and adjusted by using R version 3.6.3 in the RStudio environment, and the C-index and AUC were also calculated in the test cohort to further evaluate the performance of the nomogram.

### 2.3. Ethical Approval

SEER data are deidentified before release and do not contain any personally identifying information. As the data are publicly available, no ethical approval is required. We received permission to access the research data file in the SEER program from the National Cancer Institute, USA (reference number 12151-Nov2019).

## 3. Results

### 3.1. Demographic and Clinical Characteristics of MTC Patients

A total of 1,237 patients registered with MTC who underwent total thyroidectomy and neck lymph nodes dissection between 2004 and 2015 were enrolled from the SEER database based on the inclusion and exclusion criteria, and all patients were divided into the training group (*n* = 867) and test group (*n* = 370). Their basic information on demographic and clinical characteristics is listed in Table 1. In the training cohort, the number of patients in the training group aged <48, 48–71, and >71 was 299, 468, and 100, respectively. There were 67 black people, 740 white people, and 60 other races (American Indians, Alaska Natives, and Asian/Pacific Islanders). Female and male patients were 479 and 388, respectively. The number of patients with solitary and multiple nodules was 589 and 278. Those with tumor size <20, 20–40, and >40 mm were, respectively, 381, 318, and 168. The number of people with clinical staging stage of T1a, T1b, T2, T3a, T3b, T4a, and T4b was 168, 175, 204, 118, 125, 52, and 25. The number of patients with No, N1a, and N1b status was 391, 169, and 307. Those with metastatic status M0 and M1 were, respectively, 810 and 57. The number of those people with 1–3 or >3 cervical regional lymph nodes removed was 158 and 709, respectively. The number of MTC patients with <12, 12–32, and >32 regional lymph nodes by pathological findings was 367, 253, and 247. The number of 0, 1–16, and >16 positive lymph nodes was 393, 378, and 96. The number of those with LNR ratio <19%, 19%–49%, and >49% was 514, 191, and 162, respectively (Table 1). The information of variables in the test cohort is shown in Table 1, and the proportion of each variable is basically identical with the training cohort. In the training cohort, except for race, clinical T status, surgery scope of regional lymph nodes, the number of regional lymph nodes by pathological findings, and other variables were significantly meaningful (*P* < 0.05) in the univariate Cox regression analysis for OS. Categorical variables defined as having a significant association with OS on univariate analysis were studied in a Cox proportional hazards model, and there are four significant meaningful predictors: tumor size, age, metastasis status, and LNR in this multivariate Cox regression for OS (Table 2). In the univariate Cox regression analysis for CSS, the following seven variables were found to be significantly associated with the CSS of the primary cohort: age, sex, tumor size, clinical T status, metastasis status, the number of positive lymph nodes (pLNs), and LNR were significantly meaningful (*P* < 0.05). In the multivariate Cox regression for CSS, the following 4 variables were identified to construct the Cox proportional hazards regression model, namely, tumor size, age, metastasis status, and LNR (Table 3).

### 3.2. Development and Validation of the Nomograms for OS and CSS

**Figure 2:**
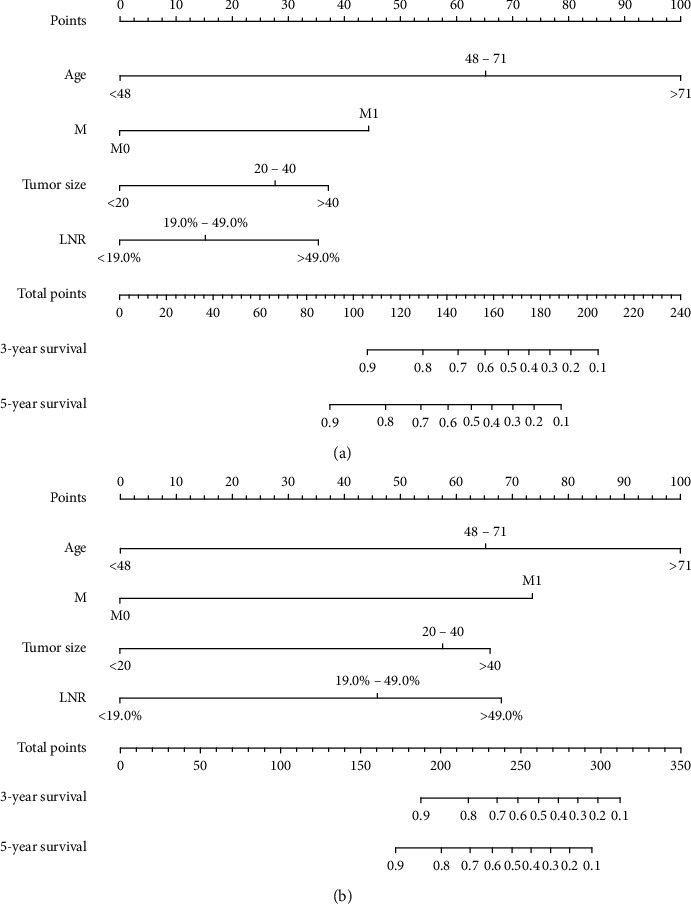
Nomograms to predict 3- and 5-year overall survival (a) and cancer-specific survival (b) for MTC patients who underwent total thyroidectomy and neck lymph nodes dissection.

**Figure 3:**
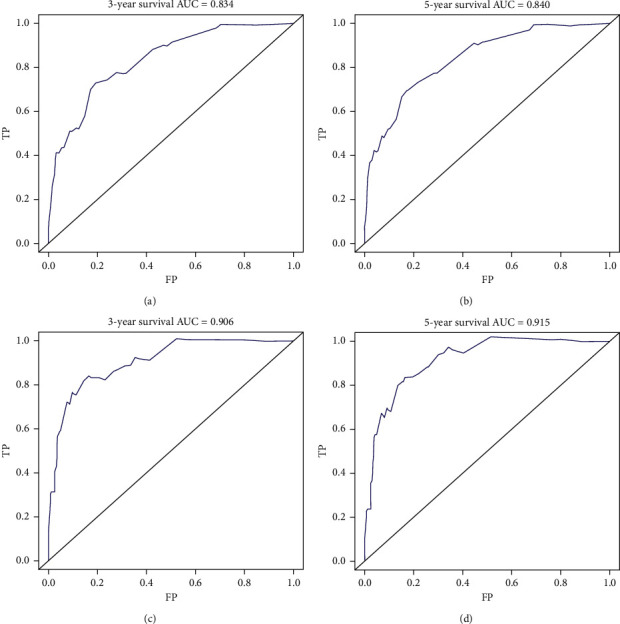
Receiver operating characteristic curves (ROC) for assessing the performance of predicting 3‐year (a) and 5‐year (b) overall survival and 3‐year (c) and 5‐year (d) cancer-specific survival in the training group.

**Figure 4:**
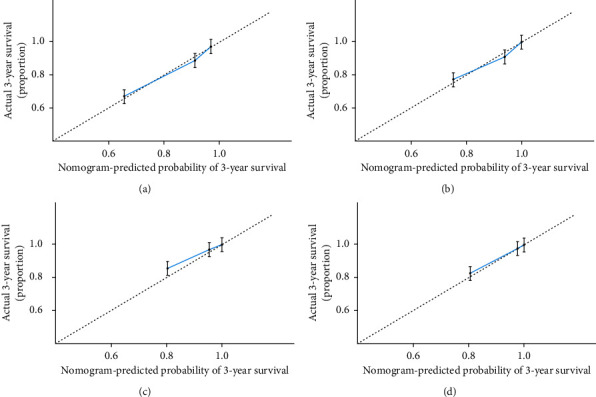
3-year (a) and 5-year (b) overall survival nomogram internal verification curves and 3-year (c) and 5-year (d) cancer-specific survival nomogram internal verification curves.

**Figure 5:**
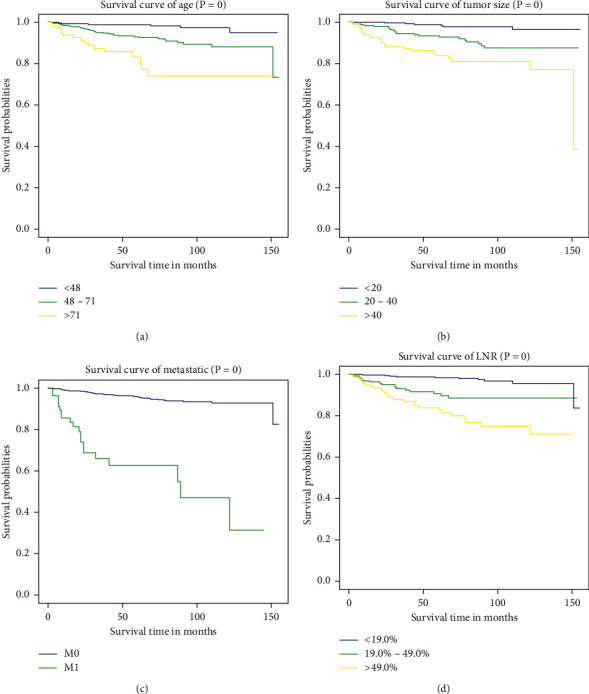
Kaplan–Meier survival curves of meaningful independent predictors: (a) age, (b) tumor size, (c) metastasis status, and (d) LNR about cancer-specific survival (CSS) in the training cohort.

**Figure 6:**
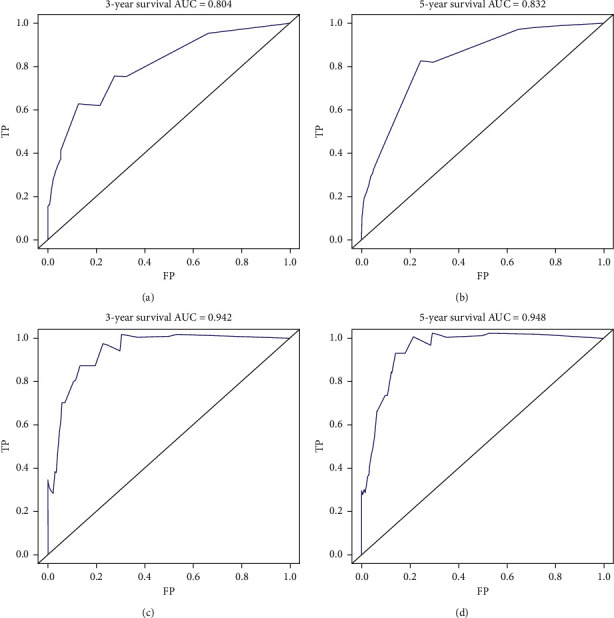
Receiver operating characteristic curves (ROC) for evaluating the performance of predicting 3‐year (a) and 5‐year (b) overall survival and 3‐year (c) and 5‐year (d) cancer-specific survival in the test cohort.

The nomograms of CSS and OS were formulated in the training cohort based on the results of multivariate analysis and the Cox proportional hazards regression model, and some nonsignificant predictors and variables with minor effects were excluded. Finally, tumor size, age, metastasis status, and LNR were selected to construct the nomogram of OS as having the highest predictive accuracy. Tumor size, age, metastasis status, and LNR were used to develop the nomogram for CSS according to the multivariate analysis outcomes. We performed internal validation to correct the bias of overfitting outcomes from testing on the same patient population. The C-index was applied to evaluate the prediction accuracy of the nomogram, and the C-index of OS and CSS was, respectively, 0.828 and 0.904, representing a relatively satisfactory predictive accuracy. The ROC is plotted in Figure 2, the 3-year survival AUC of the nomogram was 0.834 and 0.906 in OS and CSS, and the 5-year survival AUC of the nomogram was 0.84 and 0.915 in OS and CSS (Figure 3). The calibration curve demonstrated that the values of 3- and 5-year OS and CSS predicted by the nomogram were consistent with actual outcomes (Figure 4). Kaplan–Meier methods were used to analyze the survival curve of meaningful independent predictors of CSS in the training cohort (Figure 5). The external validation cohort was applied to evaluate the predictive performance of the nomogram, and the demographic information and clinical characteristics of the test cohort are listed in Table 1; the C-index was 0.828 and 0.813 for CSS and OS in the test cohort, the ROC is plotted in Figure 6, the AUC of 3- and 5- year survival was 0.942 and 0.948 in CSS, and the AUC of 3- and 5- year survival was 0.804 and 0.832 in OS. The validation of both nomograms demonstrates satisfactory agreement with predicted values.

## 4. Discussion

MTC accounts for 3%–5% of thyroid cancers worldwide. The proportion of MTC dropped to 1%–2% due to a significant increase in the relative incidence of PTC in the United States during the past three decades [1]. It is a rare histological subtype with poor prognosis compared with PTC and FTC, more than 50% of MTC patients are deemed with recurrence through biopsy examination after surgery within 10 years, and distant metastases occur in 10% of patients despite locoregional control [4, 9]. This study found that the extent of surgical resection is an independent predictor of the survival outcome for MTC patients, and primary surgical regions less than total thyroidectomy and the decreased survival rate were related. Therefore, total thyroidectomy and cervical lymph nodes dissection based on the clinical and pathologic results became a standard surgical treatment method for patients with sporadic or hereditary MTC according to the latest guideline of ATA and NCCN [1, 3].

The X-tile software has been used to find out the optimal cutoff points of continuous variables that affect the prognosis of tumor survival [10]. In the current study, the X-tile software calculated the ages of 48 and 71 as the optimal cutoff points to distinguish survival rates of MTC patients. Similarly, the optimal cutoff values of tumor size were 20 and 40 mm, and 12 and 32 regional lymph node dissection was turning point in the survival rate. The optimal cutoff points for the number of positive lymph nodes (pLNs) were 1 and 16. The best cutoff points for LNR were 19% and 49% according to the results of X-tile analysis. After the COX univariate and multivariate regression, we designed a model that systematically considers multiple variables based on the independent predictor of CSS and OS including age, tumor size, metastasis status, and LNR.

Age is an independent predictor for MTC as confirmed by many studies [11]. Patients aged >65 had a decreased survival rate and the risk of death increases by 5.2% for each additional year [3]. A previous study found that tumor size >4 cm had a significantly worse survival outcome compared to those with a diameter of ≤4 cm. The results of this study verified the previously established conclusions that lesion size is associated with a decreased survival trend [12, 13]. Distant metastases are often found in involved organs and metastases could occur in multiple organs such as lungs, liver, and bone simultaneously [2]. Compared with patients whose lesions are confined to the gland, patients with regional metastases have a 2.69 times higher risk of disease-related death, while the patients with distant metastases have a 4.47 times higher risk of disease-related death [3]. The 5-year survival rate after the diagnosis of distant metastasis was 26%, and the 10-year survival rate was 10% according to the result of a retrospective study [11, 14]. The positive rate of lymph nodes (LNR) is the ratio of the number of positive lymph nodes (pLNs) to the total number of lymph nodes examined by pathology; it has been recognized as an independent prognostic factor for the survival of breast cancer, pancreatic cancer, gastric cancer, colon cancer, uterine, and ovarian cancer. Jiang et al. [15] demonstrated that LNR could predict survival outcomes and provide guidance and suggestions on the prognosis after an operation in patients with stage IV MTC and the optimal cutoff value of LNR to predict OS was 76.5%.

Nomograms have been widely used in clinical works as a reliable predictive tool, and it solves the complexity of balancing different factors through statistical modeling so that patients and doctors can quantify risk based on the chart. At present, nomograms are used in the prognosis analysis of various malignant tumors, such as thyroid, breast, and prostate [16, 17]. Memorial Sloan-Kettering Center conducted a clinical statistical analysis of 249 MTC patients from 1986 to 2010. Allen S. Ho et al. concluded that age, gender, postoperative calcitonin, perivascular invasion, pathologic T status, pathologic N status, and M status have the highest prediction accuracy for MTC-specific mortality. The final nomogram with a C-index of 0.77 was constructed based on the seven variables [4]. The limitation of this retrospective study is that the data collection sample size is too small, resulting in controversy for this nomogram. A study that included 1,252 MTC patients with active follow-up from 1973 to 2002 in the SEER database found that age at diagnosis and stage of disease were the strongest predictors for survival [3]. However, nomogram was not constructed in that study.

We construct the nomogram based on the four prognostic factors mentioned above. To our knowledge, this is the first nomogram with excellent predictive performances to investigate the prognostic value of MTC patients who underwent total thyroidectomy and cervical dissection. The utility of the nomograms was illustrated as follows: the point score of each factor can be calculated separately by reading the score above the factor vertically, for example, 65 points above the age of 48–71 and 45 points above M1 status in the OS nomogram. The total point score was derived and then read vertically downwards to the 3- or 5-year survival HR. Taking a hypothetical patient as an example, a 50-year-old patient with a tumor size of 30 mm who underwent the standard surgical procedure was described in this article. His postoperative LNR was 50%, and there was no distant metastasis. It can be inferred that his 5-year OS is around 70% and 5-year CSS is close to 80% from the nomogram in the article. In our study, to build a refined nomogram, some controversial factors such as radiotherapy and chemotherapy were not taken into account. Treatment with radioactive iodine(I^131^) is meaningless on account of medullary carcinoma cells that originated from C cells that do not absorb I^131^. A single-center experience indicates that adjuvant radioisotope therapy was not sensitive to the medullary carcinoma cells unless the MTC patient was accompanied by histological subtypes of papillary or follicular carcinoma [18]. Besides, previous studies based on the SEER database showed that 57% of all MTC patients who received adjuvant radioisotope treatment had extrathyroidal invasion and local infiltration and found that the addition of radiation will reduce the survival rate even for patients with regional and distant metastasis [3]. Similarly, cytotoxic chemotherapy treatment has little effect on MTC [19]. As for the efficacy of newer tyrosine kinase inhibitors, more clinical trials are needed. The information on patients' adjuvant chemotherapy and molecular targeted therapy was not collected by the SEER database.

The strength of our study is that there were a large number of cases included, and the observation and follow-up time was longer. More influencing predictors were evaluated by an appropriate statistical analysis method, and a model with clinical application value was constructed based on these independent predictors. The variables of our nomogram are easily available in clinical data. The prognostic nomogram for MTC patients after total thyroidectomy and neck lymph nodes dissection was constructed because thyroid resection is the only established curative method for MTC. The nomogram performed well in predicting the survival of patients after surgery, and its prediction was supported by the C-index and the calibration curve. It can provide clinicians with a quantitative tool to assess the probability of a patient dying from local medullary thyroid carcinoma and the risk of a patient dying from a competitive death risk, such as other tumors, and also provide reference for clinicians to choose treatment options for postoperative patients. Of course, our research also has limitations. First, our study failed to include a biochemical evaluation with serum calcitonin and carcinoembryonic antigen (CEA) levels owing to SEER's lack of information regarding biochemical examination results, both of them are recognized as independent factors which predict the outcome. Meanwhile, our nomogram did not include calcitonin and CEA doubling times. Second, a classic surgical approach was used for cervical lymph nodes including unilateral or bilateral central lymphadenectomy, modified lymphadenectomy, and radical lymphadenectomy. We cannot evaluate the effectiveness of particular surgical techniques on account of this level of surgical detailed information that is not available in the SEER. Finally, familial MTC, multiple endocrine neoplasia (MEN)-2A or 2B, genetic RET mutation status, and other information about familial MTC cannot be obtained in the SEER database, which prevents us from independently assessing any predictor related to it. In addition, what we should know is SEER database derived from a variety of sites without a single standardised management protocol and from numerous surgeons with presumably varying skills, so that the outcome represents average but not necessarily optimal management. It should be noted that our nomograms have not been validated external datasets, and it is necessary to use other databases for calibration in the future. We anticipate physicians to use nomograms to evaluate prognosis, combined with clinical variables such as calcitonin, CEA, or the latest research results such as tumor necrosis and high mitotic rate [20] and expect them to confirm the output of the tool.

In summary, this study used routine clinical data to build the first nomogram model of 3- and 5-year CSS and OS among MTC patients who underwent total thyroidectomy and neck lymph nodes dissection. The nomogram provides a convenient prognostication model for the clinical practice of the surgeon and facilitates patient counseling on clinical prognosis and follow-up.

## Figures and Tables

**Table 1:** Baseline demographic and clinical characteristics of MTC patients who underwent total thyroidectomy and neck lymph nodes dissection.

Variables	Training cohort (%) (*n* = 867)	Test cohort (%) (*n* = 370)
*Age (y)*		
<48	299 (34.5)	137 (37.0)
48–71	468 (54.0)	184 (49.7)
>71	100 (11.5)	49 (13.3)

*Race*		
Black	67 (7.7)	28 (7.6)
White	740 (85.4)	319 (86.2)
Others	60 (6.9)	23 (6.2)

*Sex*		
Male	388 (44.8)	141 (38.1)
Female	479 (55.2)	229 (61.9)

*Number of nodules*		
Solitary	589 (67.9)	260 (70.3)
Multiple	278 (32.1)	110 (29.7)

*Tumor size(mm)*		
<20	381 (43.9)	162 (43.8)
20–40	318 (36.7)	151 (40.8)
>40	168 (19.4)	57 (15.4)

*T*		
T1a	168 (19.4)	83 (22.4)
T1b	175 (20.2)	77 (20.8)
T2	204 (23.5)	92 (24.9)
T3a	118 (13.6)	48 (13.0)
T3b	125 (14.4)	42 (11.4)
T4a	52 (6.0)	17 (4.6)
T4b	25 (2.9)	11 (3.0)

*N*		
N0	391 (45.1)	200 (54.1)
N1a	169 (19.5)	48 (13.0)
N1b	307 (35.4)	122 (33.0)

*M*		
M0	810 (93.4)	354 (95.7)
M1	57 (6.6)	16 (4.3)

*Number of removal LNs*		
1–3	158 (18.2)	70 (18.9)
≥4	709 (81.8)	300 (81.1)

*Total LNs*		
0–11	367 (42.3)	178 (48.1)
12–32	253 (29.2)	89 (24.1)
33–90	247 (28.5)	103 (27.8)

*PLNs*		
0	393 (45.3)	200 (54.1)
1–16	378 (43.6)	130 (35.1)
17–73	96 (11.1)	40 (10.8)

*LNR*		
<19.0%	514 (59.3)	250 (67.6)
19.0%–49.0%	191 (22.0)	60 (16.2)
>49.0%	162 (18.7)	60 (16.2)

Number of removal LNs, number of removal lymph nodes; total LNs, the number of regional lymph nodes by pathological findings; pLNs, positive lymph nodes; LNR, lymph node ratio.

**Table 2:** Univariate and multivariate cox regression analysis of overall survival (OS) in the training cohort.

Characteristics	Univariate analysis			Multivariate analysis		
	HR	95% CI	*P* value	HR	95% CI	*P* value
*Age (y)*						
<48	Reference			Reference		
48–71	6.965	3.363–14.425	≤0.001^*∗*^	7.635	3.675–15.863	≤0.001^*∗*^
>71	21.564	10.108–46.003	≤0.001^*∗*^	22.406	10.384–48.346	≤0.001^*∗*^

*Race*						
Black	Reference					
White	0.764	0.421–1.388	0.377	—	—	—
Others	0.574	0.215–1.531	0.268	—	—	—

*Sex*						
Male	Reference					
Female	0.571	0.402–0.811	0.002^*∗*^	—	—	—

*Number of nodules*						
Solitary	Reference					
Multiple	0.620	0.410–0.939	0.024^*∗*^	—	—	—

*Tumor size(mm)*						
<20	Reference			Reference		
20–40	2.429	1.512–3.902	≤0.001^*∗*^	2.369	1.468–3.824	≤0.001^*∗*^
>40	5.347	3.338–8.567	≤0.001^*∗*^	3.178	1.948–5.186	≤0.001^*∗*^

*T*						
T1a	Reference					
T1b	1.362	0.597–3.106	0.463	—	—	—
T2	2.050	0.976–4.309	0.058	—	—	—
T3a	3.505	1.659–7.404	≤0.001^*∗*^	—	—	—
T3b	4.787	2.337–9.808	≤0.001^*∗*^	—	—	—
T4a	9.006	4.236–19.148	≤0.001^*∗*^	—	—	—
T4b	6.743	2.736–16.615	≤0.001^*∗*^	—	—	—

*N*						
N0	Reference					
N1a	2.452	1.500–4.007	≤0.001^*∗*^	—	—	—
N1b	3.017	1.966–4.628	≤0.001^*∗*^	—	—	—

*M*						
M0	Reference			Reference		
M1	6.117	3.984–9.392	≤0.001^*∗*^	3.936	2.488–6.227	≤0.001^*∗*^

*Number of removal LNs*						
1–3	Reference					
≥4	1.117	0.709–1.762	0.632	—	—	—

*Total LNs*						
0–11	Reference					
12–32	1.268	0.820–1.960	0.285	—	—	—
33–90	1.645	1.088–2.486	0.018^*∗*^	—	—	—

*pLNs*						
0	Reference					
1–16	2.357	1.551–3.584	≤0.001^*∗*^	—	—	—
17–63	4.246	2.557–7.050	≤0.001^*∗*^	—	—	—

*LNR*						
<19.00%	Reference			Reference		
19.00%–49.00%	2.092	1.326–3.301	0.002^*∗*^	1.603	1.006–2.554	0.047^*∗*^
>49.00%	4.441	2.966–6.649	≤0.001^*∗*^	3.006	1.974–4.577	≤0.001^*∗*^

HR, hazard ratio; CI, confidence interval; ^*∗*^means *P* < 0.05.

**Table 3:** Univariate and multivariate cox regression analysis of cancer-specific survival (CSS) in the training cohort.

Characteristics	Univariate analysis			Multivariate analysis		
	HR	95% CI	*P* value	HR	95% CI	*P* value
*Age (y)*						
<48	Reference					
48–71	4.017	1.685–9.557	0.002^*∗*^	4.967	2.058–11.992	≤0.001^*∗*^
>71	10.827	4.223–27.761	≤0.001^*∗*^	13.397	5.004–35.863	≤0.001^*∗*^

*Race*						
Black	Reference					
White	0.843	0.336–2.115	0.715	—	—	—
Others	0.231	0.027–1.975	0.181	—	—	—

*Sex*						
Male	Reference					
Female	0.573	0.337–0.973	0.039^*∗*^	—	—	—

*Number of nodules*						
Solitary	Reference					
Multiple	0.843	0.472–1.507	0.565	—	—	—

*Tumor size (mm)*						
<20	Reference					
20–40	5.022	2.053–12.287	≤0.001^*∗*^	4.476	1.813–11.048	≤0.001^*∗*^
>40	11.455	4.712–27.846	≤0.001^*∗*^	5.558	2.223–13.895	≤0.001^*∗*^

*T*						
T1a	Reference					
T1b	1.060	0.149–7.529	0.953	—	—	—
T2	4.081	0.881–18.904	0.072	—	—	—
T3a	5.536	1.150–26.655	0.033^*∗*^	—	—	—
T3b	13.180	3.014–57.640	≤0.001^*∗*^	—	—	—
T4a	29.048	6.518–129.457	≤0.001^*∗*^	—	—	—
T4b	27.548	5.691–133.349	≤0.001^*∗*^	—	—	—

*N*						
N0	Reference					
N1a	5.847	2.388–14.313	≤0.001^*∗*^	—	—	—
N1b	7.443	3.236–17.122	≤0.001^*∗*^	—	—	—

*M*						
M0	Reference					
M1	12.854	7.382–22.384	≤0.001^*∗*^	6.694	3.690–12.145	≤0.001^*∗*^

*Number of removal LNs*						
1–3	Reference					
≥4	1.015	0.520–1.981	0.965	—	—	—

*Total LNs*						
0–11	Reference					
12–32	1.295	0.665–2.518	0.447	—	—	—
33–90	1.79	0.958–3.343	0.068	—	—	—

*pLNs*						
0	Reference					
1–16	5.737	2.506–13.131	≤0.001^*∗*^	—	—	—
17–63	11.917	4.823–29.444	≤0.001^*∗*^	—	—	—

*LNR*						
<19.00%	Reference					
19.00%–49.00%	4.536	2.117–9.720	≤0.001^*∗*^	3.287	1.493–7.235	0.003^*∗*^
>49.00%	9.875	4.868–20.034	≤0.001^*∗*^	5.874	2.794–12.352	≤0.001^*∗*^

## Data Availability

All data were generated from the SEER database. These data can be found at https://seer.cancer.gov/data/.
